# Ellagic Acid Mitigates Lead (Pb)-Induced Toxicity in *Cyprinus carpio*: A Multi-Biomarker Assessment of Hematological, Immunological, and Oxidative Stress Responses Supported by Molecular Docking

**DOI:** 10.3390/antiox15081020

**Published:** 2026-08-15

**Authors:** Mücahit Eroğlu, Mehmet Nuri Cakmak, Ayşegül Pala, Harun Uslu, Serpil Mişe Yonar, Ünal İspir, Cemal Orhan, Muhammet Enis Yonar

**Affiliations:** 1 Department of Basic Sciences, Faculty of Fisheries, Firat University, 23119 Elazig, Türkiye; meroglu44@firat.edu.tr; 2 Department of Aquaculture, Faculty of Fisheries, Firat University, 23119 Elazig, Türkiye; mncakmak@firat.edu.tr (M.N.C.); serpilmise@gmail.com (S.M.Y.); 3 Department of Aquaculture, Faculty of Fisheries, Munzur University, 62000 Tunceli, Türkiye; aysegulsvm@gmail.com; 4 Department of Pharmaceutical Chemistry, Faculty of Pharmacy, Firat University, 23119 Elazig, Türkiye; huslu@firat.edu.tr; 5 Department of Aquaculture, Fisheries Faculty, Malatya Turgut Özal University, 44210 Malatya, Türkiye; unal.ispir@ozal.edu.tr; 6 Department of Animal Nutrition, Faculty of Veterinary Medicine, Firat University, 23119 Elazig, Türkiye; corhan@firat.edu.tr

**Keywords:** lead toxicity, ellagic acid, oxidative stress, immune response, molecular docking

## Abstract

Lead (Pb) is a widespread environmental pollutant that induces systemic toxicity in aquatic organisms primarily through oxidative stress, hematological disruption, and immune dysfunction. This study investigated the protective effects of ellagic acid (EA) against Pb-induced toxicity in common carp (*Cyprinus carpio*) using a multi-biomarker approach and molecular docking. Fish were assigned to six experimental groups: control, EA-treated, Pb-I, Pb-I + EA, Pb-II, and Pb-II + EA. Fish in the Pb-I and Pb-II groups were exposed to 2.5 and 5 mg/L Pb, respectively, while EA was administered via diet at 100 mg/kg for 14 days. At the end of the exposure period, hematological indices, innate immune parameters, and oxidative stress biomarkers were assessed in blood, liver, kidney, and gill tissues. Pb exposure caused marked hematological impairment, suppressed immune responses, increased malondialdehyde levels, and disrupted antioxidant defense by reducing SOD, CAT, GSH-Px, and GSH levels while increasing GST activity. In contrast, dietary EA supplementation significantly mitigated Pb-induced alterations, improved hematological and immunological responses, reduced lipid peroxidation, restored antioxidant capacity, and normalized GST activity to control levels. Molecular docking analyses further showed that EA interacts with hemoglobin and immunoglobulin M, supporting its potential role in preserving oxygen transport and immune functions under Pb-induced stress. Overall, the findings demonstrate that Pb-induced toxicity involves coordinated disruption of redox homeostasis and immune function, whereas EA exerts a multi-target protective effect through biochemical and molecular mechanisms. This study provides mechanistic insight into chemical–biological interactions and supports the potential application of natural bioactive compounds in mitigating heavy metal-induced toxicity.

## 1. Introduction

Heavy metals are toxic environmental pollutants that pose significant risks to ecosystems and living organisms. While some trace metals, such as copper (Cu), zinc (Zn), iron (Fe), and manganese (Mn), play roles in biochemical processes, lead (Pb) is a non-essential metal with no known biological function and can be highly toxic even at low concentrations [1]. Pb exposure in fish, due to its high affinity for erythrocytes, disrupts membrane structure and function, thereby increasing susceptibility to oxidative stress. Additionally, metal exposure alters immunological parameters and is closely associated with changes in the immune system. Pb is also recognized as a significant immunotoxin in animals [2]. The effects of Pb exposure have been extensively investigated in various fish species, demonstrating its impact on tissue accumulation, behavior, hematological and immunological responses, and antioxidant defense systems [3,4,5,6,7,8,9]. Oxidative stress caused by reactive oxygen species (ROS) occurs when antioxidant defenses are insufficient. Exposure to toxic substances in aquatic organisms increases ROS such as superoxide anion, hydrogen peroxide, and hydroxyl radicals, leading to cellular damage. Changes in antioxidant levels serve as reliable biomarkers of toxicity. Pb is a significant contributor to oxidative stress [10].

Natural botanical compounds are increasingly used to enhance the performance of cultured aquatic species. These compounds are typically incorporated into feeds to promote growth, improve overall health, strengthen the immune system, and increase resistance to disease [11]. Among these compounds, ellagic acid (EA) is a naturally occurring polyphenol widely distributed in fruits and nuts, including pomegranate, strawberry, raspberry, grape, blackberry, and walnut [12]. EA is known for its strong antioxidant properties, as it can scavenge free radicals, chelate metal ions, and modulate oxidative processes under physiological conditions. Moreover, its metabolites further contribute to its biological activity, enabling effective protection against oxidative stress even at relatively low concentrations [13]. Despite the well-documented antioxidant potential of EA and the known toxic effects of Pb, studies investigating their combined effects remain limited. In particular, the extent to which EA can mitigate Pb-induced hematological, immunological, and oxidative alterations in economically important fish species remains unclear. Although the antioxidant, immunomodulatory, and cytoprotective effects of EA have been demonstrated in various experimental models [14,15], studies in fish have primarily focused on growth performance and antioxidant defense [11,12]. While the beneficial effects of EA on hematological, immunological, and antioxidant parameters have been reported in *Cyprinus carpio* and other fish species, and its protective role against certain toxic agents has been documented [16,17,18,19], investigations addressing its effects under heavy metal toxicity, particularly Pb exposure, remain limited.

Therefore, the present study aimed to comprehensively evaluate the protective and ameliorative effects of EA against sublethal Pb exposure in *C. carpio*. To this end, hematological, immunological, oxidative stress, and antioxidant parameters were assessed simultaneously to elucidate both the multifaceted impact of Pb toxicity and the regulatory role of EA in mitigating these alterations. By adopting a multi-biomarker approach, this study provides integrative insight into the protective responses induced by EA against Pb-induced toxicity, thereby offering a novel contribution to the existing literature.

## 2. Material and Methods

### 2.1. Ethics Statement

All experimental procedures were conducted in accordance with ethical standards and approved by the Animal Experiments Ethics Committee of Munzur University (MUAEEC, Tunceli, Türkiye) under protocol number 22-06 (15-05).

### 2.2. Lead (Pb)

In the experiments, Pb was administered as lead nitrate (Pb(NO_3_)_2_; CAS No. 10099-74-8) from Sigma-Aldrich (Burlington, MA, USA).

### 2.3. Ellagic Acid

EA was obtained from Sigma-Aldrich Chemical Co. (St. Louis, MO, USA). All chemicals and reagents used in the study were of analytical grade. Due to its limited solubility under natural conditions, EA was first dissolved in an alkaline solution of 0.01 M NaOH (approximately pH 12). After EA was added to the NaOH solution, the pH of the mixture decreased to approximately 8, as previously described. The resulting solution (pH ≈ 8) was then incorporated into the experimental diet. The NaOH solution was used solely as a solvent to facilitate EA dissolution during diet preparation and did not constitute an experimental treatment.

### 2.4. Preparation of Ellagic Acid-Supplemented Diet

Commercial basal feed was ground into a fine powder and thoroughly mixed with a prepared EA solution to achieve a final concentration of 100 mg EA/kg diet. The mixture was then re-pelleted and dried at room temperature. The resulting pellets were stored at 4 °C until use. The amount of NaOH added during diet preparation was minimal and used solely to facilitate EA dissolution before feed incorporation.

The selected EA dose was based on previous studies reporting its safety and biological effectiveness in fish [16,17,18,19]. The pellets were manually administered to the fish twice daily at a feeding rate of approximately 2% of their body weight throughout the experiment.

### 2.5. Fish and Experimental Design

Common carp (*C. carpio*) with an average body weight of 44.71 ± 3.10 g were obtained from a local fish farm and transported alive to the laboratory. Upon arrival, fish were transferred to 100 L glass aquaria with continuous aeration. Prior to the experiment, all individuals were visually inspected, and only clinically healthy fish with no external signs of disease, injury, or abnormal behavior were selected. All experimental fish were obtained from the same commercial supplier, selected from a single stock population, and acclimated under identical laboratory conditions. Following the acclimation period, fish were randomly assigned to experimental groups to minimize variation associated with individual or environmental background factors.

Fish were acclimated to laboratory conditions for one month before the experiment. During this period, aquaria were filled with dechlorinated tap water, with dissolved oxygen levels of 7.7 ± 0.5 mg/L and a pH of 7.05 ± 0.15. Water temperature was maintained at 23.5 ± 1 °C under a 12 h light/12 h dark photoperiod. Water quality parameters were regularly monitored throughout both the acclimation and experimental periods. Following acclimation, fish were randomly assigned to six experimental groups. Each treatment group consisted of three tanks, with 10 fish per tank, for a total of 30 fish per treatment group. During the chronic exposure experiment, fish were maintained collectively within three tanks per treatment group. Each treatment group represented individual biological observations from three tanks (*n* = 30). Therefore, the tanks served as the experimental exposure environment, while individual fish measurements were recorded as biological responses within that shared exposure condition. Hematological, immunological, and oxidative stress biomarkers were measured separately for each sampled fish.

The experimental groups were:

- Control group: Fish were maintained under standard conditions without treatment.
- EA group: Fish were fed a diet supplemented with 100 mg of EA per kg of diet.
- Pb-I group: Fish were exposed to 2.5 mg/L of Pb.
- Pb-I + EA group: Fish were exposed to 2.5 mg/L Pb and fed an EA-supplemented diet simultaneously.
- Pb-II group: Fish were exposed to 5 mg/L of Pb.
- Pb-II + EA group: Fish were exposed to 5 mg/L Pb and fed an EA-supplemented diet.

The experiment was conducted under semi-static conditions for 14 days. Under this exposure regime, the aquarium water was renewed every other day, and freshly prepared Pb solutions were added to restore the nominal exposure concentrations while maintaining stable water quality throughout the experimental period. Pb exposure was delivered via the waterborne route. Fish were monitored daily, and no mortality was observed during the experimental period.

The applied Pb concentrations were set to sublethal levels corresponding to ½ and ¼ of the LC_50_ value (10 mg/L) reported in the literature [20].

### 2.6. Sampling and Sample Preparation

At the end of the 14-day experimental period, fish were anesthetized with benzocaine, and blood samples were collected via a caudal peduncle incision. Blood samples were divided into K_3_EDTA tubes for hematological and immunological analyses and into plain tubes for serum separation.

Whole blood was analyzed on the same day for red blood cells (RBC), white blood cells (WBC), hemoglobin (Hb), hematocrit (Ht), and erythrocyte indices (MCV, MCH, MCHC). Oxidative radical production (NBT) and phagocytic activity (PA) were also determined. Serum was obtained after clotting at room temperature for 30 min and centrifugation at 5000× *g* for 10 min, then stored at −20 °C for analyses of total protein (TP), immunoglobulin M (IgM), lysozyme (LYZ), and myeloperoxidase (MPO) levels.

After blood collection, fish were dissected, and liver, kidney, and gill tissues were collected. Tissues were rinsed with 0.9% NaCl and stored at −80 °C until analysis. Samples were homogenized in 1.15% KCl (1:10, *w*/*v*) and centrifuged at 18,000× *g* for 30 min at 4 °C [21]. Supernatants were used to determine Malondialdehyde (MDA) and glutathione (GSH) levels, as well as the activities of superoxide dismutase (SOD), catalase (CAT), glutathione peroxidase (GSH-Px), and glutathione S-transferase (GST).

### 2.7. Assessment of Hematological Parameters

RBC counts were determined using a hemocytometer (Isolab, Wertheim, Germany) after dilution with Natt and Herrick solution [22]. Hb concentration was measured spectrophotometrically at 540 nm with Drabkin’s reagent. Ht values were obtained by the microhematocrit centrifugation method. Based on the measured RBC, Hb, and Ht values, erythrocyte indices—including mean corpuscular volume (MCV), mean corpuscular hemoglobin (MCH), and mean corpuscular hemoglobin concentration (MCHC)—were calculated using standard formulas [23,24].

### 2.8. Assessment of Innate Immune Responses

WBC count was determined simultaneously with RBC count using the method described by Yonar et al. [22]. NBT activity, an indicator of the respiratory burst capacity of phagocytic cells, was determined using the protocol described by Siwicki et al. [25]. PA, reflecting the ability of phagocytic cells to engulf foreign particles, was determined using the method described by Siwicki et al. [26]. TP and IgM levels were determined using the procedures described by Siwicki et al. [25] and Siwicki et al. [27], respectively. LYZ activity, an indicator of innate antibacterial defense, was determined using the procedure described by Ellis [28]. MPO activity, an indicator of neutrophil and phagocyte activity, was measured using the method of Quade and Roth [29], with minor modifications reported by Sahoo et al. [30].

### 2.9. Assessment of Oxidative Stress Biomarkers and Antioxidant Enzyme Activities

Lipid peroxidation (LPO) was quantified by measuring malondialdehyde (MDA) levels using the thiobarbituric acid (TBA) reaction method described by Placer et al. [31]. Tissue GSH concentrations were measured using the procedure of Ellman [32]. Antioxidant enzyme activities were evaluated spectrophotometrically: SOD activity was determined following Sun et al. [33]; CAT activity was measured using the method of Aebi [34]; GSH-Px activity was assessed according to Beutler [35]; and GST activity was analyzed using the protocol reported by Habig et al. [36]. The activities of antioxidant enzymes (SOD, CAT, GST, and GSH-Px), as well as MDA and GSH levels, were normalized to the protein concentration of tissue homogenates, which was determined using the method of Lowry et al. [37].

### 2.10. Molecular Docking Studies

The SMILES data for the EA ligand were retrieved from the PubChem website (https://pubchem.ncbi.nlm.nih.gov/), plotted using ChemDraw (ChemOffice Ultra 8.0.3), and energy minimization was performed. PDB files of macromolecules were obtained from the Protein Data Bank (https://www.rcsb.org/), and Maestro Release 2026-1 (version 14.7) was modified and optimized using [38]. The grid box was set to 60 Å3 with 0.375 Å spacing, consistent with previously determined or predicted active-site regions for fish Hb (PDB ID: 3BOM) and IgM (PDB ID: 8GHZ) [39,40]. 50 runs were performed for Hb and IgM using standard settings as in our previous studies [41]. The Lamarckian Genetic Algorithm was used in all AutoDock studies, and both AutoDock (version 1.5.7) [42] and Vina (version 1.1.2) [43] results are presented in detail in Table 1 and Section 3. As a result of the validation procedure, RMSD values were measured as 0.21 for HEM in the Hb macromolecule.

### 2.11. Statistical Analyzes

Statistical analyses were performed using GraphPad Prism [version 11.0.0(84); GraphPad Software Inc., San Diego, CA, USA]. For hematological, immunological, and oxidative stress biomarkers, individual fish measurements served as biological observations because blood and tissue samples were collected and analyzed separately for each fish. Data are presented as mean and standard deviation; *n* = 30 represents the total number of individually sampled fish per treatment group. Normality of the data distribution was assessed using the Shapiro–Wilk test, and homogeneity of variances was evaluated using the Bartlett test prior to parametric analysis. Differences among experimental groups were analyzed using one-way analysis of variance (ANOVA). When a significant overall effect was detected, Tukey’s multiple-comparison post hoc test was applied to determine differences between group means. Statistical significance was accepted at *p* < 0.05.

## 3. Results

### 3.1. Hematological Findings

**Figure 1:**
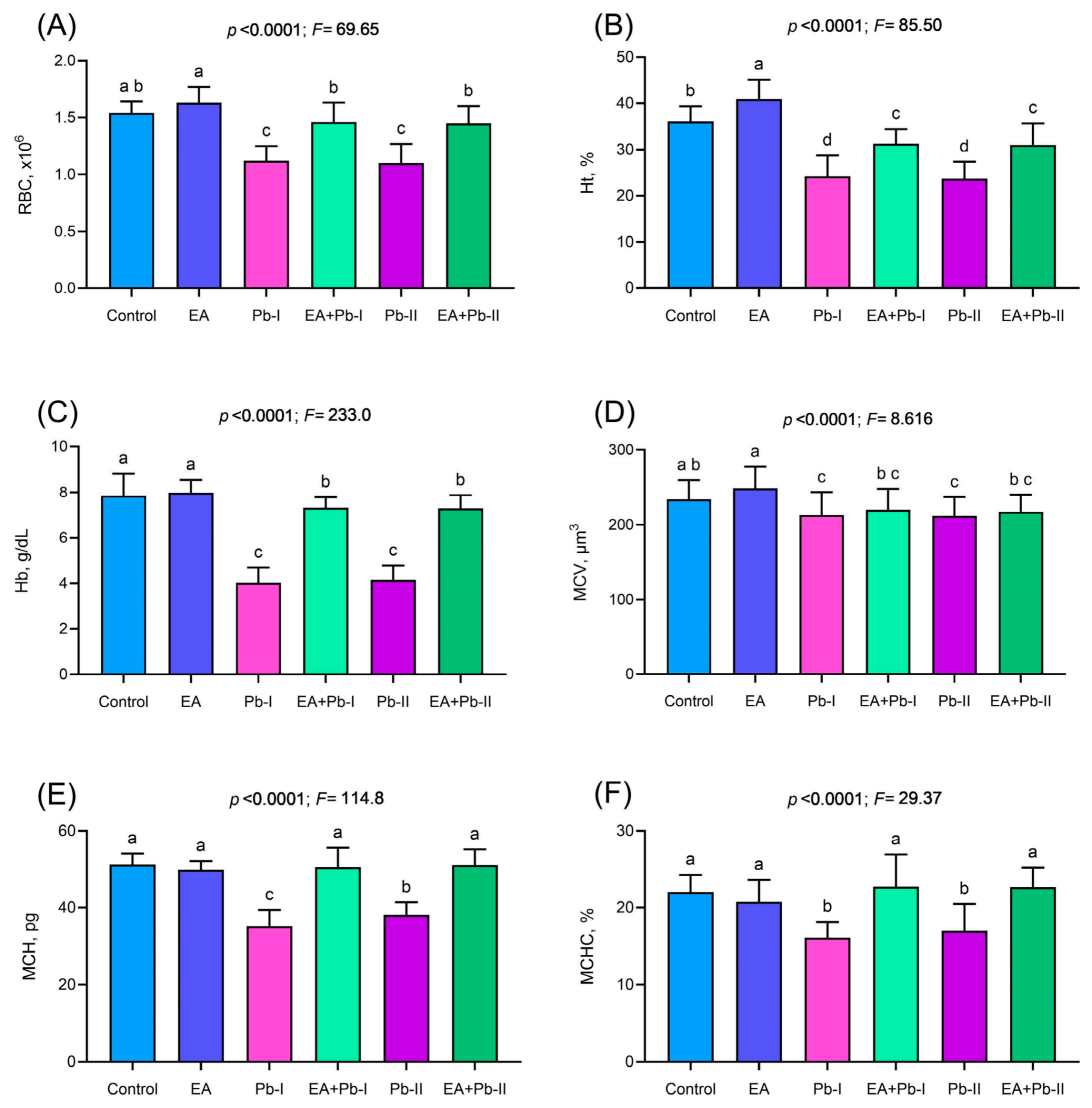
The effects of ellagic acid (EA) supplementation on carp (*Cyprinus carpio*) exposed to lead (Pb) toxicity were evaluated based on hematological parameters [RBC: Red blood cell (erythrocyte) count (**A**), Hb: Hemoglobin concentration (**B**), Ht: Hematocrit level (**C**), MCV: Mean corpuscular volume (**D**), MCH: Mean corpuscular hemoglobin (**E**), and MCHC: Mean corpuscular hemoglobin concentration (**F**)]. Fish were exposed to lead at 2.5 and 5.0 mg/L, and ellagic acid was supplemented at 100 mg/kg diet. Data are presented as mean ± standard deviation; *n* = 30 denotes the total number of individually sampled fish per treatment group. Statistically significant differences among experimental groups are indicated by different superscript letters (a–d), as determined by one-way analysis of variance (ANOVA) followed by Tukey’s multiple comparison test (*p* < 0.05).

Exposure to both Pb-I and Pb-II concentrations resulted in significant reductions in all evaluated hematological indices compared to the control group (RBC, Hb, Ht, MCV, MCH, MCHC) (*p* < 0.05). In contrast, fish receiving only EA supplementation exhibited higher RBC counts and Ht values than the control group, while the remaining hematological parameters did not differ significantly. In the groups co-treated with Pb and EA, most hematological parameters were significantly improved relative to the Pb-only groups (*p* < 0.05); however, MCV remained statistically unchanged (*p* > 0.05) (Figure 1).

### 3.2. Immunological Findings

**Figure 2:**
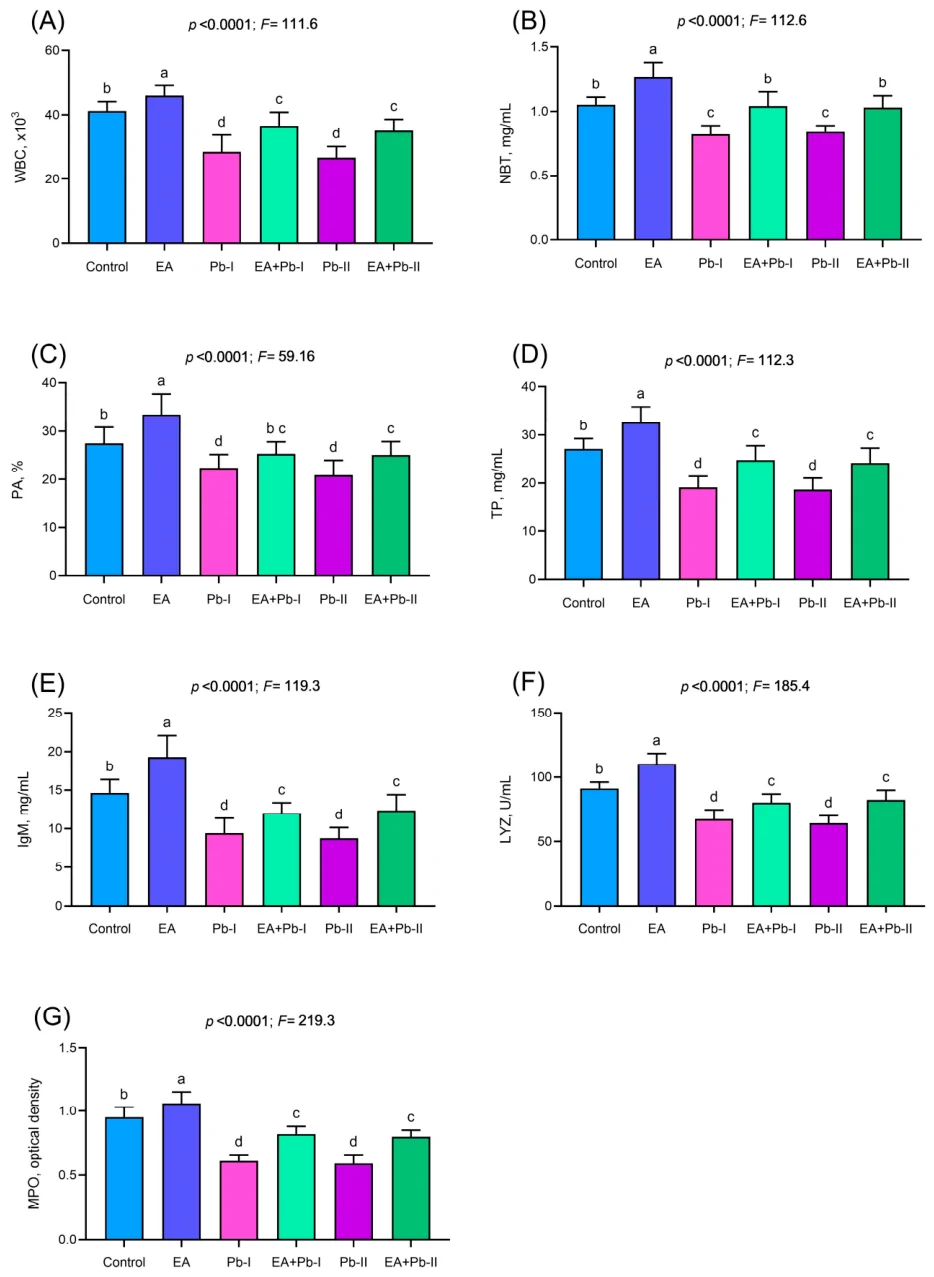
The effects of ellagic acid (EA) supplementation on carp (*Cyprinus carpio*) exposed to lead (Pb) toxicity were evaluated based on immunological parameters [WBC: White blood cell count (**A**), NBT: Nitroblue tetrazolium test (**B**), PA: Phagocytic activity (**C**), TP: Total protein level (**D**), IgM: Total immunoglobulin M level (**E**), LYZ: Lysozyme activity (**F**), and MPO: Myeloperoxidase activity (**G**)]. Fish were exposed to lead at 2.5 and 5.0 mg/L, and ellagic acid was supplemented at 100 mg/kg diet. Data are presented as mean ± standard deviation; *n* = 30 denotes the total number of individually sampled fish per treatment group. Statistically significant differences among experimental groups are indicated by different superscript letters (a–d), as determined by one-way analysis of variance (ANOVA) followed by Tukey’s multiple comparison test (*p* < 0.05).

Exposure to Pb resulted in significant reductions in non-specific immune parameters. In the Pb-I and Pb-II groups, WBC, NBT, PA, TP, IgM, LYZ, and MPO levels were significantly lower than those in the control group (*p* < 0.05). In contrast, EA supplementation alone significantly increased all evaluated immunological parameters compared to the control (*p* < 0.05). Moreover, co-administration of EA with Pb significantly elevated these immune indices relative to the Pb-only groups (*p* < 0.05) (Figure 2).

### 3.3. Oxidative Stress and Antioxidant Parameters

**Figure 3:**
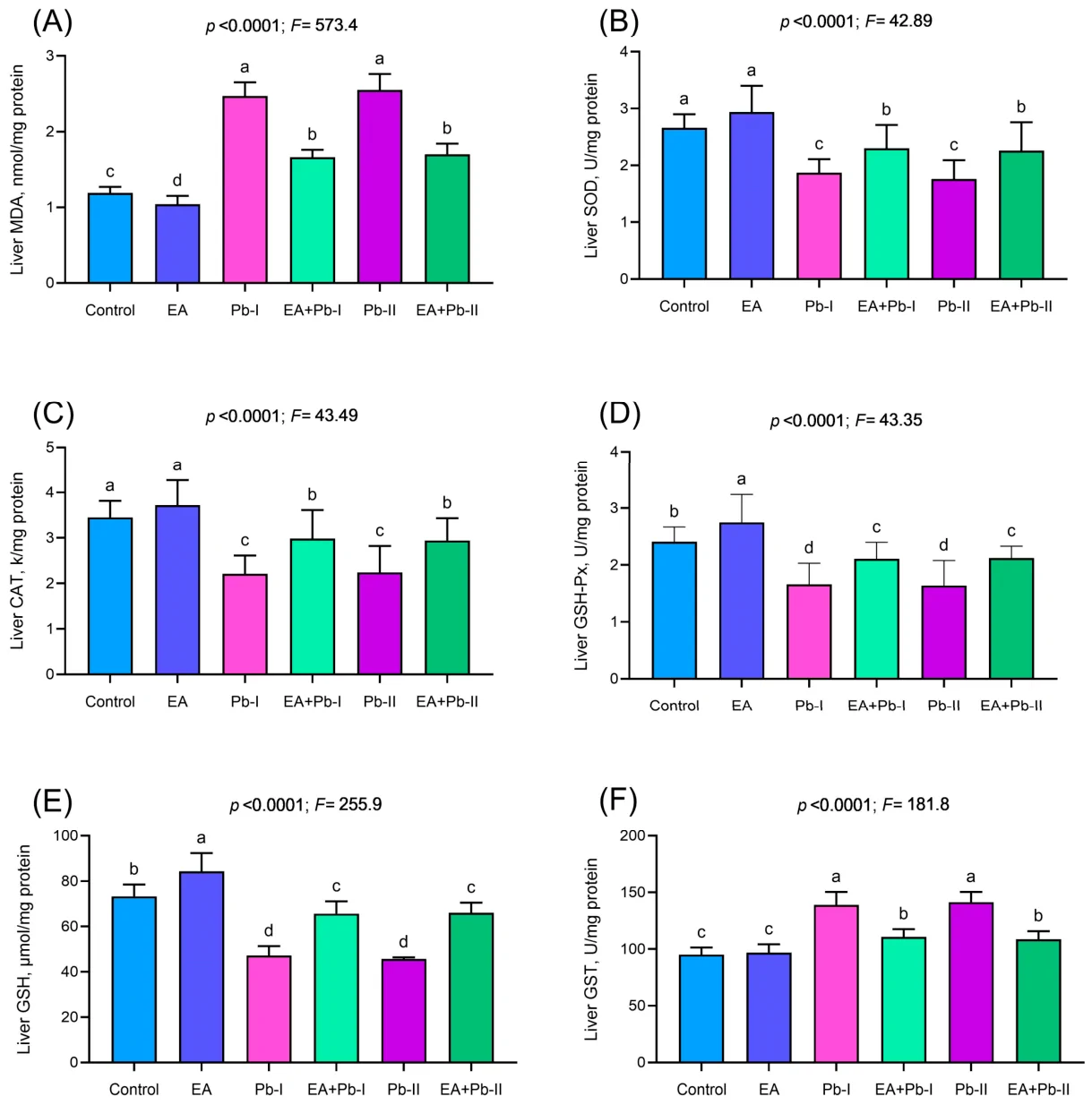
The effects of ellagic acid (EA) supplementation on carp (*Cyprinus carpio*) exposed to lead (Pb) toxicity were evaluated based on oxidative stress [MDA: Malondialdehyde (**A**)] and antioxidant parameters [SOD: Superoxide dismutase activity (**B**), CAT: Catalase activity (**C**), GSH-Px: Glutathione peroxidase activity (**D**), GSH: Reduced glutathione level (**E**), and GST: Glutathione-S-transferase activity (**F**)] in the liver. Fish were exposed to lead at 2.5 and 5.0 mg/L, and ellagic acid was supplemented at 100 mg/kg diet. Data are presented as mean ± standard deviation; *n* = 30 denotes the total number of individually sampled fish per treatment group. Statistically significant differences among experimental groups are indicated by different superscript letters (a–d), as determined by one-way analysis of variance (ANOVA) followed by Tukey’s multiple comparison test (*p* < 0.05).

**Figure 4:**
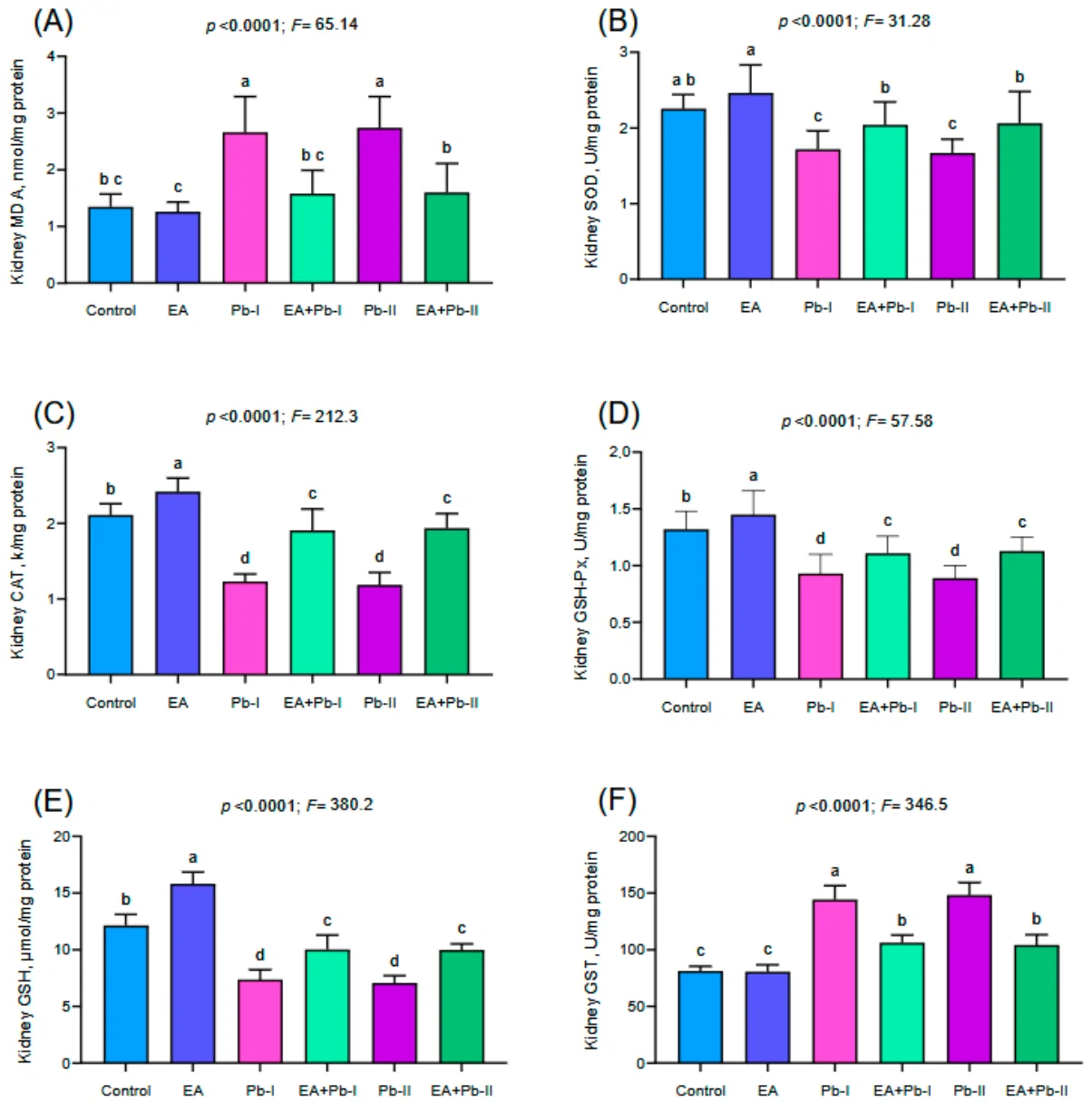
The effects of ellagic acid (EA) supplementation on carp (*Cyprinus carpio*) exposed to lead (Pb) toxicity were evaluated based on oxidative stress [MDA: Malondialdehyde (**A**)] and antioxidant parameters [SOD: Superoxide dismutase activity (**B**), CAT: Catalase activity (**C**), GSH-Px: Glutathione peroxidase activity (**D**), GSH: Reduced glutathione level (**E**), and GST: Glutathione-S-transferase activity (**F**)] in the kidney. Fish were exposed to lead at 2.5 and 5.0 mg/L, and ellagic acid was supplemented at 100 mg/kg diet. Data are presented as mean ± standard deviation; *n* = 30 denotes the total number of individually sampled fish per treatment group. Statistically significant differences among experimental groups are indicated by different superscript letters (a–d), as determined by one-way analysis of variance (ANOVA) followed by Tukey’s multiple comparison test (*p* < 0.05).

**Figure 5:**
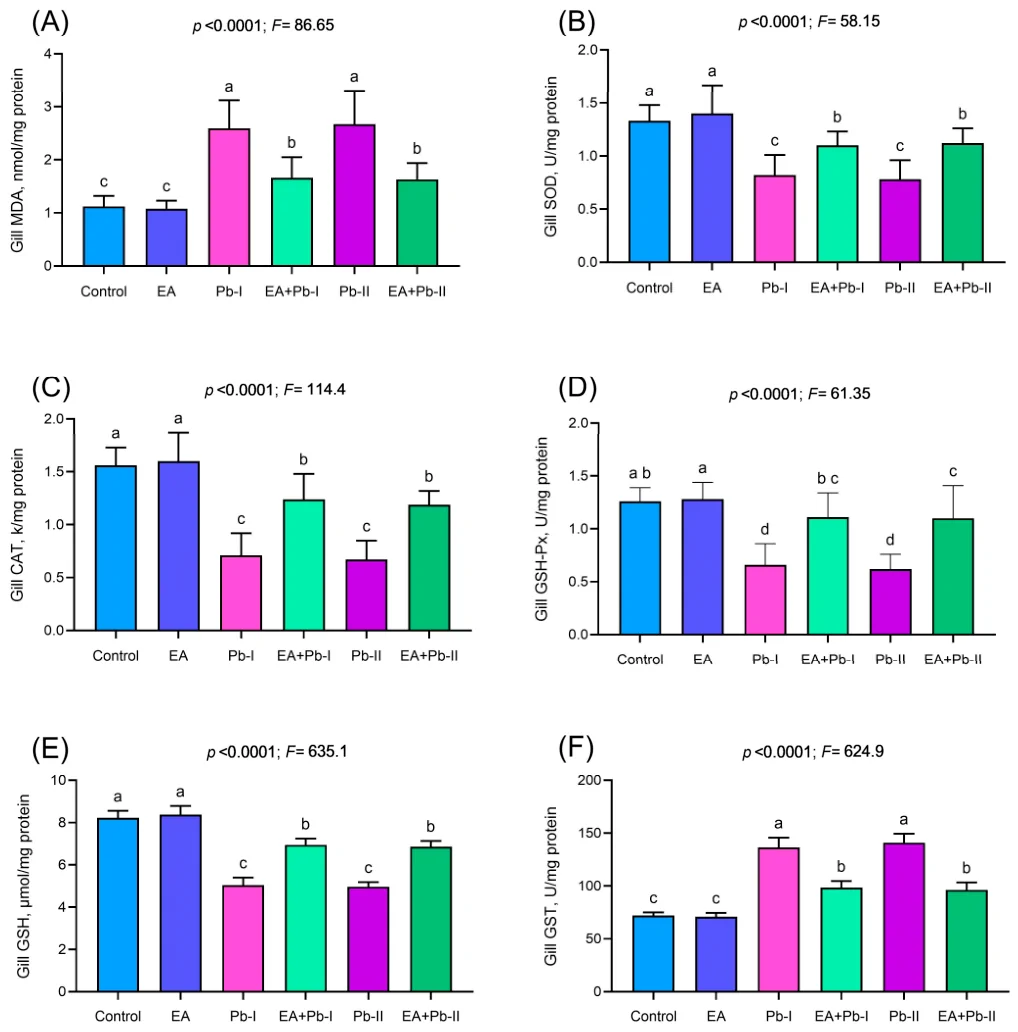
The effects of ellagic acid (EA) supplementation on carp (*Cyprinus carpio*) exposed to lead (Pb) toxicity were evaluated based on oxidative stress [MDA: Malondialdehyde (**A**)] and antioxidant parameters [SOD: Superoxide dismutase activity (**B**), CAT: Catalase activity (**C**), GSH-Px: Glutathione peroxidase activity (**D**), GSH: Reduced glutathione level (**E**), and GST: Glutathione-S-transferase activity (**F**)] in the gill. Fish were exposed to lead at 2.5 and 5.0 mg/L, and ellagic acid was supplemented at 100 mg/kg diet. Data are presented as mean ± standard deviation; *n* = 30 denotes the total number of individually sampled fish per treatment group. Statistically significant differences among experimental groups are indicated by different superscript letters (a–d), as determined by one-way analysis of variance (ANOVA) followed by Tukey’s multiple comparison test (*p* < 0.05).

Pb exposure markedly elevated MDA levels in the liver (Figure 3A), kidney (Figure 4A), and gill (Figure 5A) tissues (*p* < 0.05). In both Pb-I and Pb-II groups, tissue MDA concentrations were significantly higher than those recorded in the control group. In contrast, the EA-only group exhibited significantly lower MDA levels in the liver and kidney compared to the control, whereas gill MDA values did not differ from the control. Combined treatment with EA and Pb significantly attenuated MDA levels in all tissues relative to Pb exposure alone (*p* < 0.05) (Figure 3A, Figure 4A and Figure 5A).

A significant suppression of SOD activity was observed in all examined tissues (Figure 3B, Figure 4B and Figure 5B) following Pb exposure. Both Pb-I and Pb-II groups displayed significantly reduced SOD activities compared to the control (*p* < 0.05). Conversely, dietary EA supplementation alone enhanced SOD activities across all tissues. When administered concurrently with Pb, EA significantly restored SOD activity relative to the Pb-only groups (*p* < 0.05) (Figure 3B, Figure 4B and Figure 5B).

Similarly, CAT activities were significantly diminished in the liver (Figure 3C), kidney (Figure 4C), and gill (Figure 5C) tissues of Pb-exposed fish. In both Pb-treated groups, CAT values remained significantly below control levels (*p* < 0.05). Fish receiving dietary EA alone exhibited significantly higher CAT activities in the liver and kidney than the control group, whereas CAT activity in the gill did not differ significantly from the control (*p* > 0.05). Co-treatment with Pb and EA significantly increased CAT activities in all examined tissues compared with the corresponding Pb-only groups (*p* < 0.05) (Figure 3C, Figure 4C and Figure 5C).

Pb treatment also led to pronounced reductions in GSH-Px activities in all tissues examined (Figure 3D, Figure 4D and Figure 5D). Activities in the Pb-I and Pb-II groups were significantly lower than those in the control (*p* < 0.05). In contrast, EA administration alone significantly enhanced GSH-Px activities. The combined EA and Pb groups demonstrated significantly greater GSH-Px activities than the Pb-only groups (*p* < 0.05) (Figure 3D, Figure 4D and Figure 5D).

GSH levels showed a pattern consistent with the responses of the antioxidant enzymes. Pb exposure significantly decreased GSH concentrations in the liver (Figure 3E), kidney (Figure 4E), and gill (Figure 5E) tissues compared with the control group (*p* < 0.05). Fish receiving dietary EA alone exhibited significantly higher GSH levels in the liver and kidney than the control group, whereas GSH levels in the gill remained statistically unchanged (*p* > 0.05). Co-treatment with Pb and EA significantly increased GSH concentrations in all examined tissues compared with the corresponding Pb-only groups (*p* < 0.05) (Figure 3E, Figure 4E and Figure 5E).

In contrast to the other antioxidant parameters, GST activity exhibited a significant elevation in response to Pb exposure in all tissues (Figure 3F, Figure 4F and Figure 5F). Both Pb-I and Pb-II groups showed significantly higher GST activities than the control (*p* < 0.05). GST activity in the EA-only group did not differ from the control. However, co-administration of EA with Pb significantly reduced GST activity compared to Pb-only treatment (*p* < 0.05) (Figure 3F, Figure 4F and Figure 5F).

### 3.4. Molecular Docking Studies

**Figure 6:**
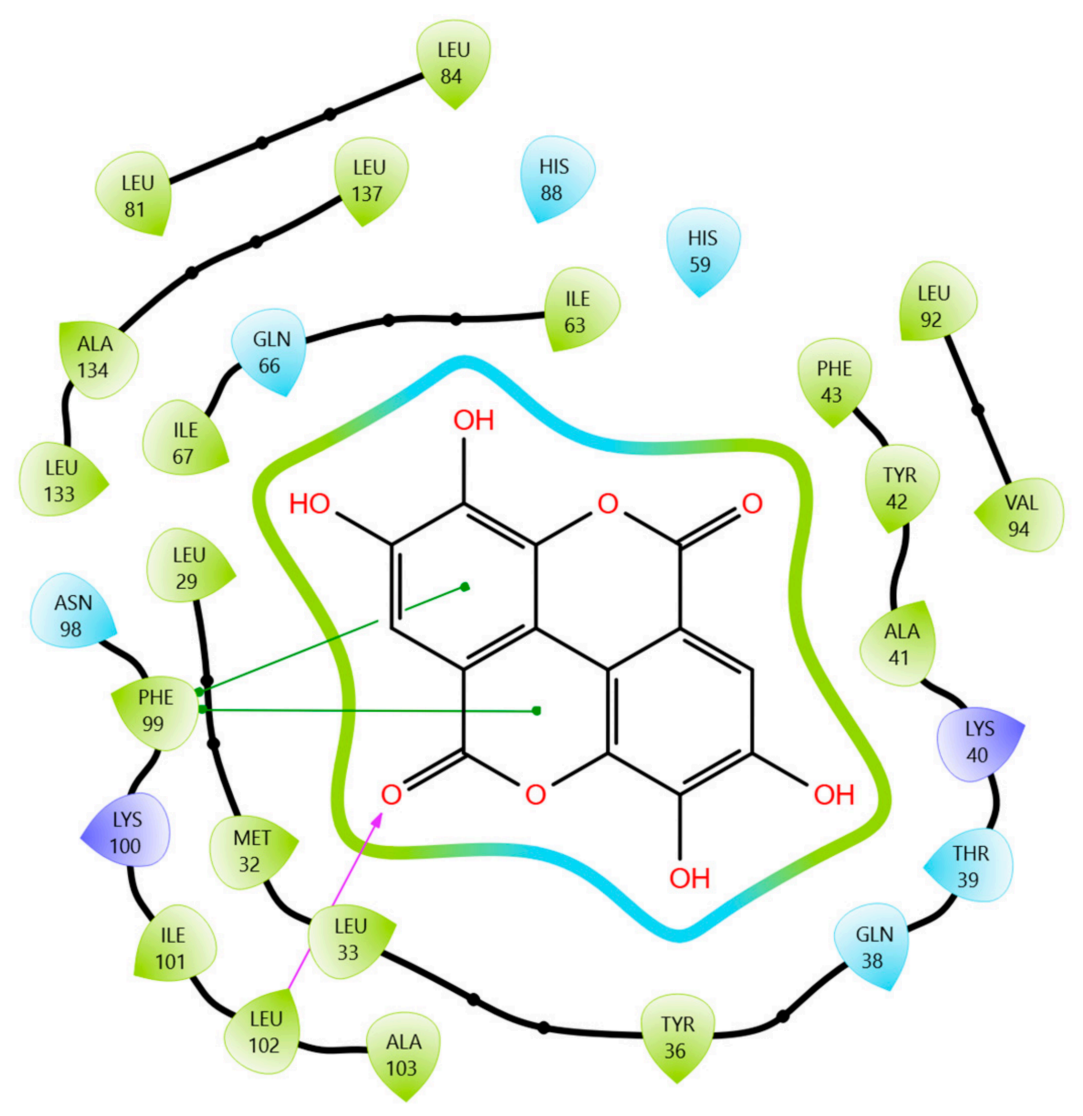
2D interaction diagram with Hb for EA.

**Figure 7:**
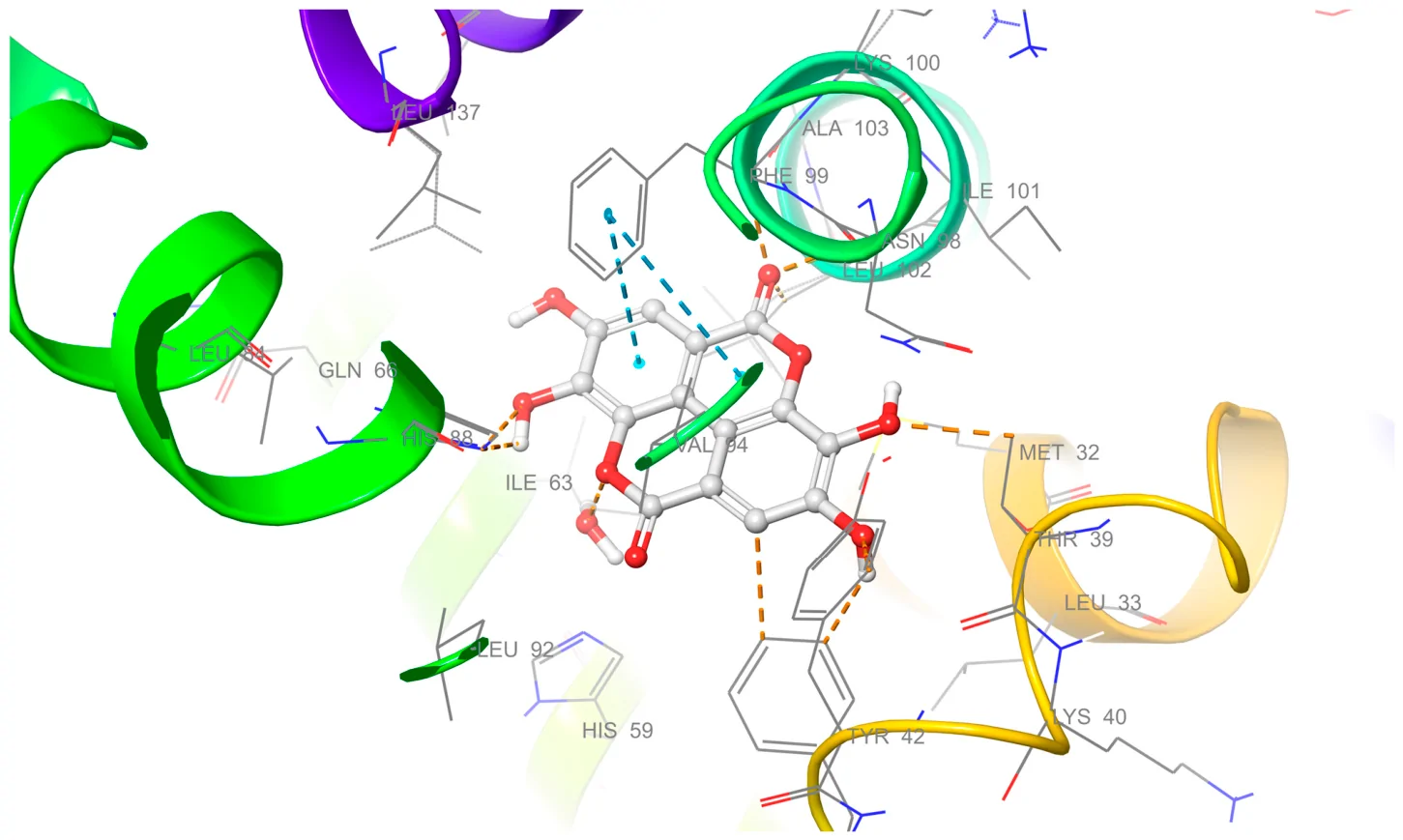
3D interaction diagram with Hb for EA.

**Figure 8:**
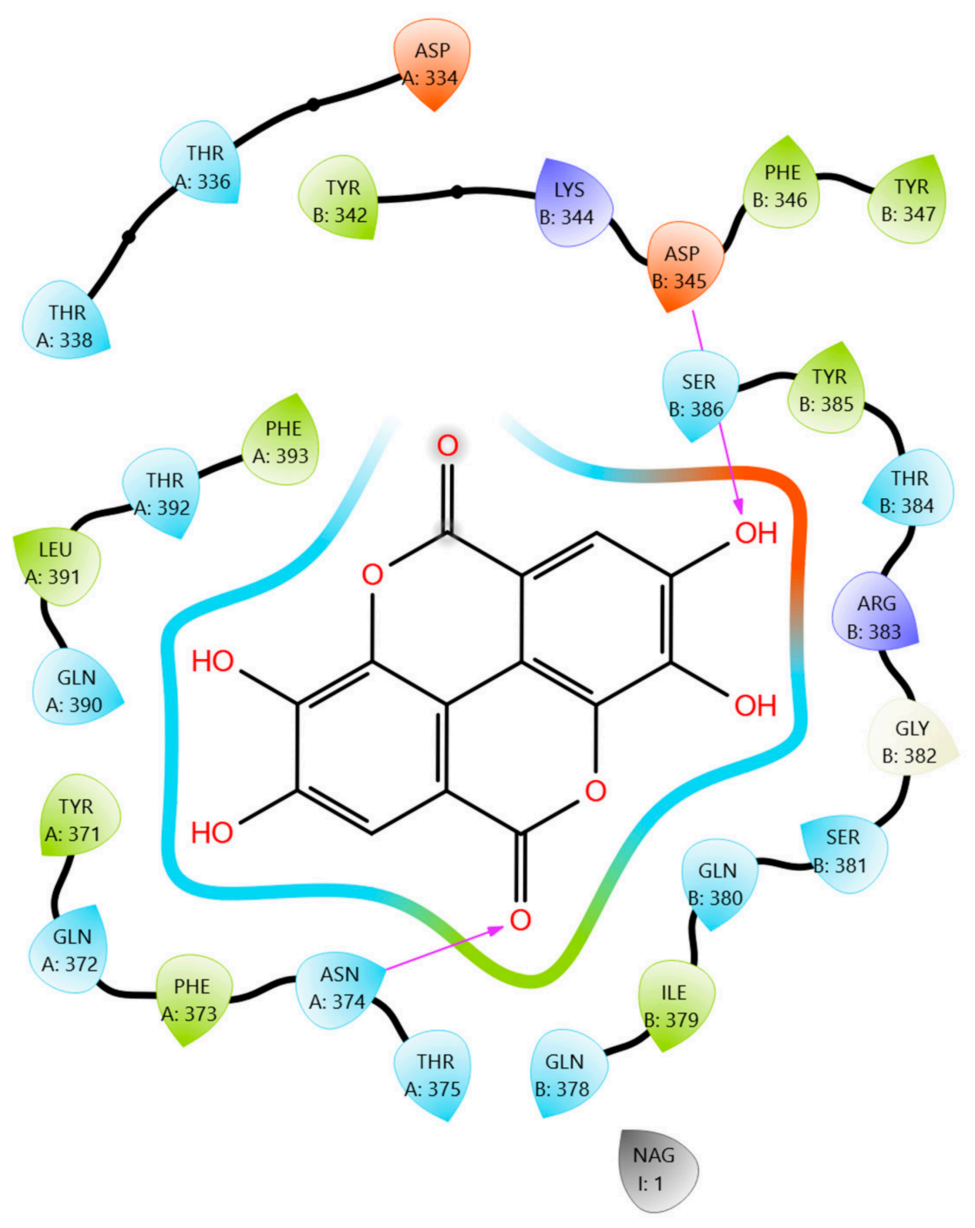
2D interaction diagram with IgM for EA.

**Figure 9:**
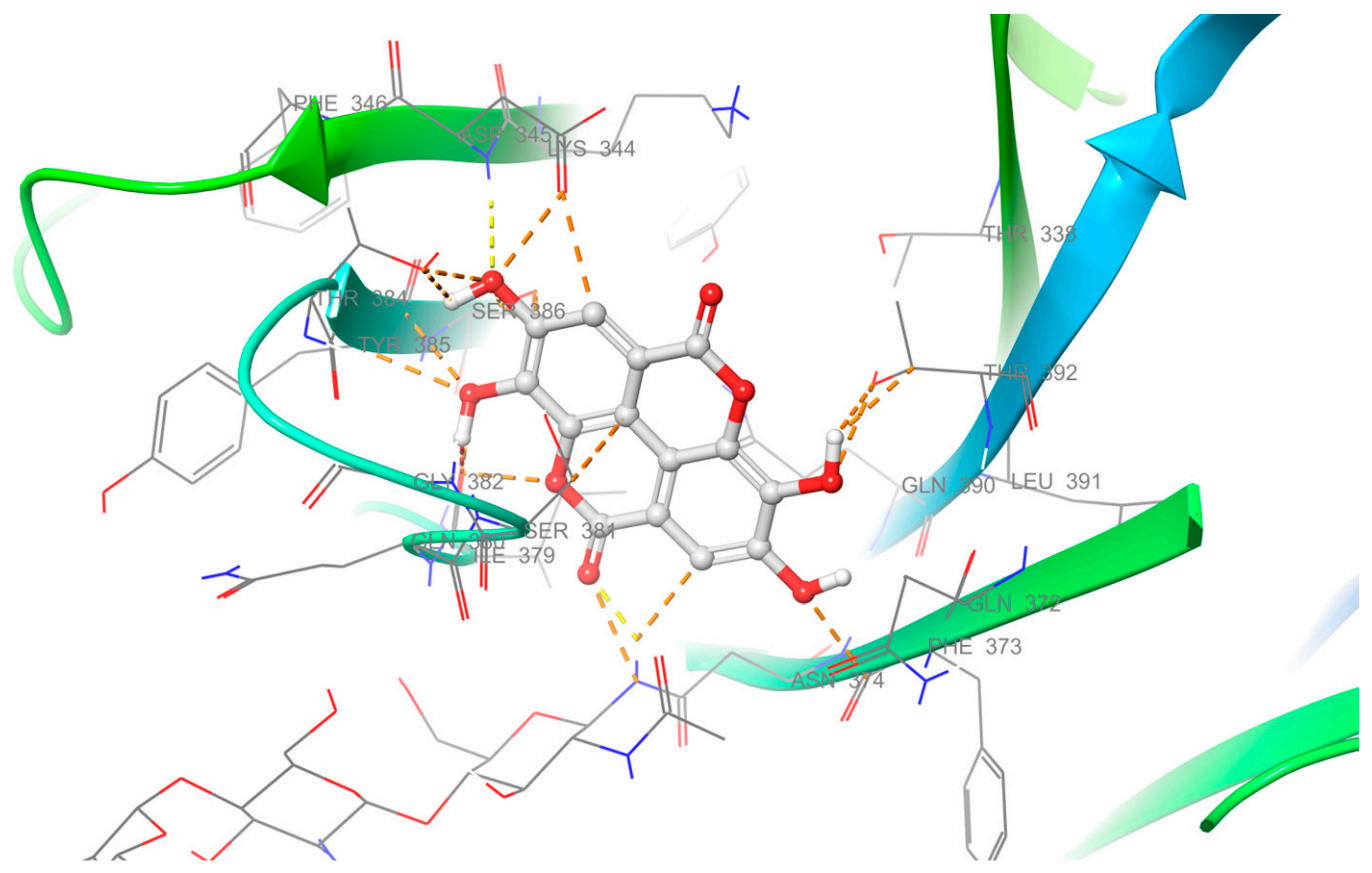
3D interaction diagram with IgM for EA.

It has been determined that EA forms hydrogen bonds with the following residue of Hb: LEU102; and also EA forms hydrogen bonds with the following residues of IgM: A:ASN374 and B:ASP345. It has been calculated that ellagic acid will exhibit micromolar binding affinity on Hb and IgM macromolecules (Table 1 and Figure 6, Figure 7, Figure 8 and Figure 9).

## 4. Discussion

In the present study, the effects of Pb exposure and dietary EA, a naturally occurring polyphenolic compound, were comprehensively evaluated by assessing hematological indices, innate immune responses, and oxidative stress biomarkers in common carp. A comparative evaluation of the Pb, EA, and Pb + EA groups demonstrated that dietary EA markedly alleviated Pb-induced physiological disturbances by improving hematological parameters, enhancing innate immune function, and restoring antioxidant defenses across multiple tissues.

Rather than demonstrating isolated protective effects on individual biomarkers, the present findings indicate that EA elicits a coordinated, multi-system protective response against Pb toxicity. The simultaneous improvement of hematological parameters, innate immune function, and antioxidant status across multiple tissues suggests that the benefits of EA extend beyond its well-known free radical-scavenging activity. Instead, the present results support the concept that EA helps preserve overall physiological homeostasis by acting on multiple interconnected biological systems during Pb-induced oxidative stress.

The blood profile is a key indicator that reflects metabolic and physiological processes in fish and also plays an important role in defense mechanisms against diseases [44]. Ht, Hb, RBC, and erythrocyte indices including mean corpuscular volume (MCV), mean corpuscular hemoglobin (MCH), and mean corpuscular hemoglobin concentration (MCHC) are fundamental hematological parameters widely used to evaluate the physiological status and health condition of fish [23]. In the present study, exposure to Pb resulted in pronounced alterations in hematological parameters in *C. carpio*, indicating a clear disruption of normal blood physiology. Significant decreases in RBC, Hb, Ht, and erythrocyte indices (MCV, MCH, and MCHC) levels were observed in Pb-exposed groups, suggesting the development of anemia. These findings are consistent with previous studies demonstrating that lead interferes with erythropoiesis and promotes erythrocyte destruction. One of the principal mechanisms underlying Pb-induced anemia is the inhibition of δ-aminolevulinic acid dehydratase (δ-ALAD), a key enzyme in the heme biosynthetic pathway. Inhibition of δ-ALAD impairs the conversion of δ-aminolevulinic acid to porphobilinogen, thereby disrupting heme synthesis and ultimately reducing hemoglobin production. Consequently, impaired erythropoiesis and the development of anemia are considered characteristic manifestations of Pb toxicity. Therefore, the decreases in Hb, RBC, and Ht observed in the present study are consistent with the well-established inhibitory effect of Pb on heme biosynthesis. This mechanism is further supported by previous studies. Vo et al. [45] reported reduced RBC, Hb, and Ht levels in red tilapia (*Oreochromis* sp.) exposed to different concentrations of Pb, attributing these changes to impaired hematopoietic activity and increased oxidative damage to erythrocyte membranes. Similarly, Batool et al. [46] documented significant declines in hematological indices (RBC, Hb and Ht levels) in *Labeo rohita*, indicating that Pb toxicity disrupts oxygen transport capacity and physiological homeostasis. These types of changes in erythrocyte indices are generally associated with macrocytic or hypochromic anemia, depending on the intensity and duration of exposure. Similar findings were also reported by Habib et al. [9], who observed significant changes in erythrocyte indices in *C. carpio* after lead exposure.

The hematological disorders induced by Pb were significantly alleviated in the fish that received the EA supplements. The RBC count, Hb content, and Ht values were significantly higher in the EA-treated fish compared with the fish that received the Pb supplements. This is an important finding, as the EA-treated fish showed normalization of the hematological disorders induced by the lead. This normalizing effect of EA on Pb-induced hematological disorders is likely associated with the antioxidant activity of EA, which may reduce oxidative damage to erythrocytes. The normalization of the erythrocyte indices, such as MCV, MCH, and MCHC, in the EA-treated fish also supports the role of the EA in the maintenance of the integrity of the erythrocytes. These observations are in line with previous studies indicating that dietary EA supplementation significantly improves hematological parameters in fish, as documented in Mişe Yonar et al. [16] and Mişe Yonar [12].

To determine the immunological effects of Pb in fish, hematological and immune-related parameters, including WBC count, NBT activity, PA, TP level, LYZ activity, MPO activity, and IgM level, were measured to assess the overall health status of the exposed fish. Leukocytes are crucial for immune regulation. Changes in WBC levels after exposure to toxic substances may signal a reduced non-specific immune response in fish [47]. The present findings show that WBC levels decreased in fish from the Pb-I and Pb-II groups. This trend aligns with previous research, such as Habib et al. [9], who documented reduced WBC counts in carp exposed to sublethal concentrations of Pb(NO_3_)_2_, and Hossain et al. [48], who reported a significant decline in WBC levels in *Oreochromis niloticus* exposed to Pb-contaminated river water. The respiratory burst is an oxygen-dependent defense mechanism of phagocytic cells, characterized by the production of reactive oxygen species. An increase in this activity is associated with enhanced pathogen-killing capacity of phagocytes [49]. In this study, respiratory burst activity was determined by the reduction in NBT by intracellular superoxide radicals produced by leukocytes, and MPO activity was also measured as an additional indicator of phagocytic activity. Pb exposure resulted in a significant decrease in NBT levels, indicating suppression of the respiratory burst capacity of phagocytic cells. Consistently, previous studies have demonstrated that lead acetate suppresses the respiratory burst activity of phagocytic cells in zebrafish larvae, reducing ROS production and consequently impairing innate immune function [50]. Similarly, findings based on oxidative burst activity (OBA) and infection assays have shown marked suppression of the immune system in *Oreochromis niloticus* exposed to Pb(NO_3_)_2_ [51]. MPO is a key component of host defense against bacterial infections in fish, and hypochlorous acid (HOCl) generated via the MPO–H_2_O_2_ system exhibits strong bactericidal activity [52]. Therefore, changes in MPO activity are considered an important indicator for evaluating the immune capacity of fish. In the present study, a 14-day exposure to Pb at concentrations of 2.5 and 5 mg/L resulted in a significant reduction in MPO activity in fish. The findings are consistent with the existing literature. In *Oreochromis mossambicus*, exposure to sublethal Pb concentrations (0.5, 2.5, and 5 mg/L) for 7 and 14 days was reported to decrease MPO activity, with reductions observed in the medium and high doses on day 7 and in all groups on day 14 [53]. Similarly, Paul et al. [6] reported that exposure of *Channa punctatus* to Pb acetate (9.43 mg/L) for four days resulted in insufficient MPO release from macrophages, which was associated with suppressed bactericidal activity and a weakened host defense mechanism.

Phagocytes are key components of the innate immune system and become particularly important when specific immune responses are suppressed, with phagocytosis serving as their primary mechanism for eliminating pathogens [54,55]. A significant decrease in PA was observed in fish after Pb exposure, consistent with the findings of Sherif et al. [56], who reported reduced PA levels in *O. niloticus* exposed to 51 µg/L Pb for 8 weeks.

Evaluation of serum biochemical parameters provides important insights into the target organs affected by toxicity and the organism’s overall health status. In addition, these parameters are considered early indicators of potential adverse changes in stressed organisms [57]. TP is widely used as a biomarker of stress and hepatic dysfunction in fish and may vary in response to environmental stressors such as Pb exposure [1]. In this study, TP levels significantly decreased in fish following Pb exposure. This finding is consistent with Ayyat et al. [58], who reported reduced serum TP levels in *O. niloticus* fed Pb-contaminated diets, and with Sahiti et al. [59], who observed a significant decline in TP levels in fish exposed to heavy metals such as Pb, Cd, and Cr compared to the control group, and attributed this decrease to protein degradation associated with stress induced by metal toxicity.

In fish, LYZ, an enzyme with antibacterial properties released by leukocytes, exhibits a broader range of activity than mammalian lysozyme. Therefore, it is commonly used as an indicator of non-specific immune responses and plays a primary role in defense against infections in fish [60]. In this study, a significant decrease in LYZ activity was observed in fish after 14 days of exposure to Pb. This supports previous studies: Dai et al. [61] found reduced LYZ activity in *C. carpio* exposed to 0.05, 0.5, and 1 mg/L Pb for 30 and 60 days, and Guo et al. [62] reported similar declines with 0, 5, 50, and 500 μg/L Pb over 7 and 14 days of exposure.

In teleost fish, IgM further mediates immune responses by binding to pathogens, thereby facilitating opsonization, triggering antibody-dependent cellular cytotoxicity, and activating the complement system, ultimately enhancing the host’s defense mechanisms [63]. The results indicate that Pb exposure suppresses IgM levels in fish, resulting in a significant decrease. Similarly, the literature reports that increasing Pb concentrations in *Channa argus* lead to reduced IgM levels, which is associated with suppression of the immune response [64]. Furthermore, exposure to 5, 50, and 500 µg/L Pb for 7, 14, and 28 days has been shown to cause a concentration-dependent decrease in IgM levels [62].

The observed decreases in WBC count, PA, TP level, NBT activity, MPO activity, LYZ activity, and particularly IgM level clearly demonstrate that Pb exposure suppresses both phagocytic activity and humoral immune responses, highlighting a pronounced immunosuppressive effect. Compared with Pb-exposed groups, EA supplementation significantly increased WBC counts, NBT activity, PA, TP, IgM levels, LYZ, and MPO activity. These findings indicate the immunostimulatory effect of EA. Improvements in phagocytic parameters suggest the preservation of cellular defense mechanisms, while increases in lysozyme and IgM levels indicate a partial restoration of humoral immune function. More importantly, in the Pb + EA groups, all parameters were markedly increased compared with the Pb groups, and most values approached those of the control group. This suggests that EA substantially ameliorates Pb-induced immunosuppression and helps restore disrupted immune homeostasis. In agreement with previous findings, fish fed EA-supplemented diets showed significantly enhanced respiratory burst activity and phagocytic capacity, confirming the enhancement of non-specific cellular immunity [12]. Similarly, EA supplementation has been reported to stimulate both specific and non-specific immune responses in rainbow trout [16], further supporting its immunomodulatory potential.

Proper physiological function in fish depends on maintaining redox homeostasis. As a result of cellular metabolism, ROS, including hydroxyl radicals, superoxide anions, nitric oxide, and hydrogen peroxide, are produced [65,66]. Fish mitigate oxidative stress by balancing the production and removal of reactive oxygen species (ROS) through their antioxidant system. This system includes enzyme antioxidants such as SOD, CAT, GST, GR, and GSH-Px, as well as non-enzyme components like GSH, metallothioneins, and vitamins C and E, which help prevent cellular damage. A decrease in antioxidant capacity and/or an excessive increase in ROS disrupts cellular balance, leading to oxidative stress [67,68,69,70]. A major component of oxidative damage is the peroxidative degradation of unsaturated fatty acids, and the resulting increase in MDA levels is widely accepted as a reliable indicator of LPO [71]. In the present study, significant increases in MDA levels were observed in fish exposed to both Pb concentrations across all examined tissues (liver, kidney, and gill). This finding indicates that Pb induces widespread LPO across different tissue types. Similarly, Yin et al. [72] reported time-dependent increases in MDA levels in zebrafish gills following exposure to sublethal concentrations of Cu, Cr, Cd, and Pb over 24–96 h, with a more pronounced response in the Pb group. In addition, Dey et al. [73] documented that long-term exposure to an environmentally relevant Pb concentration (43.3 ppm) for 15, 30, and 90 days resulted in increased MDA levels in the liver tissue of climbing perch (*Anabas testudineus*). Taken together, these findings across different exposure durations and tissue types indicate that Pb enhances lipid peroxidation under both short- and long-term conditions, which is consistent with the multi-tissue and multi-concentration responses observed in the present study. In the group treated with EA alone, the observed decrease in MDA levels, particularly in the liver and kidney tissues, indicates the antioxidant potential of this compound. Furthermore, the significant reduction in MDA levels when co-administered with Pb clearly demonstrates the suppressive and protective effect of EA against Pb-induced oxidative stress. Similar results have also been reported in the literature. Mottola et al. [14] reported that EA exerts a protective effect against H_2_O_2_-induced oxidative stress, while Xin et al. [74] demonstrated that EA reduces MDA levels by limiting LPO. These findings indicate that EA exhibits consistent protective effects under different stress conditions.

SOD, CAT, and GSH-Px are key antioxidant enzymes that protect cells from oxidative damage. SOD catalyzes the dismutation of superoxide anions (O_2_^−^) into hydrogen peroxide (H_2_O_2_), and CAT and GSH-Px subsequently detoxify hydrogen peroxide and organic peroxides. Because the integrity and function of these enzymes depend on essential trace elements, they are potential targets for lead- induced toxicity [75]. The present findings indicate that Pb exposure significantly decreased SOD, CAT, and GSH-Px activities across all examined tissues. This suggests that Pb suppresses the antioxidant enzyme system, thereby promoting oxidative stress. Similarly, Saliu et al. [76] reported that 28 days of Pb(NO_3_)_2_ exposure reduced hepatic SOD and CAT activities in fish. In addition, a study on *Clarias gariepinus* showed that 15 days of Pb exposure significantly reduced SOD and total antioxidant capacity compared with the control group [77]. These findings support the conclusion that Pb consistently impairs the antioxidant defense system across different species. In contrast, EA administration alone enhanced these enzyme activities in all examined tissues, thereby supporting the antioxidant defense system. Consistent with this, Khanzadeh et al. [11] reported that dietary supplementation with EA at different concentrations (0.10, 0.15, and 0.20 g/kg) increased SOD, CAT, and GSH-Px activities in *O. niloticus*. More importantly, co- treatment with EA significantly ameliorated the Pb- induced decreases in enzyme activities, resulting in markedly higher values than in the Pb- only groups. This improvement suggests that EA helps maintain cellular redox balance by both activating enzymatic antioxidant defenses and reducing the oxidative burden. The antioxidant improvement observed in the present study is also consistent with previous reports. Ural et al. [17] demonstrated that EA treatment alleviated malathion- related oxidative stress- induced alterations in SOD, CAT, and GSH-Px activities. Similarly, Wang et al. [15] reported that dietary EA supplementation alleviated ammonia- induced oxidative stress in *P. fulvidraco* by increasing CAT and SOD activities and total antioxidant capacity in the liver. Collectively, these findings indicate that EA exerts a protective effect against oxidative stress by strengthening the antioxidant enzyme system across different species and stress conditions.

GSH, a tripeptide antioxidant, is a fundamental defense mechanism that protects cells against ROS and their secondary damage products. Furthermore, GSH has a high affinity for metal ions [78]. In this study, Pb exposure caused a significant drop in GSH levels in the liver, kidney, and gills. This indicates that GSH is rapidly depleted during periods of increased oxidative stress. Abdel-Aziz et al. [79] also found reduced hepatic GSH in *O. niloticus*. Atiq et al. [80] reported that chronic Pb exposure led to marked declines in GSH in both liver and gill tissues. Kim and Kang [10] observed significant reductions in GSH in the liver and gills of juvenile *Sebastes schlegelii* after Pb exposure. Together, these results suggest that Pb consistently weakens antioxidant defenses by depleting GSH across various fish species. In contrast, in the groups treated with EA alone, GSH levels in the liver and kidney tissues exceeded those of the control group, indicating the strong antioxidant capacity of this compound. The higher GSH levels observed in the EA-only group compared with the control group may be associated with the intrinsic antioxidant properties of EA. In addition to scavenging reactive oxygen species, EA may help preserve intracellular GSH levels and promote endogenous GSH synthesis, thereby increasing the cellular glutathione pool even under non-stressed physiological conditions. More importantly, co-treatment with EA significantly ameliorated the Pb-induced decrease in GSH levels across all examined tissues. This improvement suggests that EA enhances cellular antioxidant defense by preserving or restoring GSH levels under oxidative stress. Similarly, previous studies have reported comparable protective effects of EA across different oxidative stress models. For instance, GSH levels in zebrafish liver were significantly reduced after iodoacetamide (IAA) exposure; however, EA supplementation prevented this depletion and maintained GSH levels, demonstrating its protective role against oxidative stress [81].

GST catalyzes the conjugation of a wide range of electrophilic xenobiotics, thereby protecting cells against their harmful effects [82]. In the present study, unlike the other antioxidant parameters, GST activity was significantly increased in all examined tissues after Pb exposure, indicating an adaptive detoxification response to Pb-induced oxidative stress. Similarly, Eroğlu et al. [83] reported increased GST activity in the liver of *Oreochromis niloticus* after exposure to 1 μg/mL Pb for 1, 7, and 14 days, with the greatest increase on day 7. In addition, Firidin [84] demonstrated that exposure of *O. niloticus* to 0.1 and 1.0 mg/L Pb for 7 and 21 days significantly elevated GST activity in both liver and kidney tissues. These findings indicate that GST exhibits a consistent response to Pb exposure across tissues and exposure durations. This differential response can be explained by the distinct physiological role of GST within the cellular defense system. Unlike SOD, CAT, and GPx, which constitute the primary enzymatic antioxidant defense responsible for scavenging reactive oxygen species, GST primarily functions as a phase II detoxification enzyme that catalyzes the conjugation of reduced glutathione with electrophilic compounds generated during oxidative stress, thereby facilitating their detoxification and elimination rather than directly scavenging reactive oxygen species. Consequently, the induction of GST activity most likely represents an adaptive cellular response to increased xenobiotic and oxidative burden rather than an enhancement of the primary antioxidant defense system. In contrast, the concomitant suppression of SOD, CAT, and GPx suggests that excessive reactive oxygen species production exceeded the capacity of the primary antioxidant defense network, whereas GST remained inducible as part of the cellular detoxification process [85,86,87]. While no significant change in GST activity was observed in the EA-only group, co-administration of EA with Pb significantly reduced GST activity compared with the Pb-only group. These findings indicate that the elevated GST activity observed after Pb exposure reflects a compensatory detoxification response to oxidative stress, whereas dietary EA reduced the requirement for this adaptive response by alleviating the oxidative burden. The obtained results are consistent with Mişe Yonar et al. [19], who reported that the increase in GST activity induced by chlorpyrifos exposure was attenuated by EA supplementation, highlighting the regulatory role of EA in detoxification and antioxidant defense. Similarly, Ural et al. [17] demonstrated that malathion exposure significantly increased tissue GST activity, whereas EA administration reduced these elevated GST levels in malathion-treated fish. Collectively, these findings suggest that EA contributes to the maintenance of cellular redox homeostasis by reducing toxin-induced oxidative stress and normalizing GST-mediated detoxification processes.

The present findings clearly demonstrate that Pb toxicity in *C. carpio* is closely associated with disruption of redox homeostasis, impairment of hematological function, and suppression of innate immunity. The ability of EA to reverse most of these alterations suggests that its protective effects are mediated by antioxidant and immunomodulatory mechanisms. By lowering MDA and GST levels, preserving GSH content, enhancing SOD, CAT, and GSH-Px activities, and improving hematological and immune indices, EA effectively reduced the physiological burden imposed by Pb. These results suggest that EA may serve as a promising nutritional strategy to improve fish resilience under heavy metal stress.

The crystal structure of fish Hb (PDB ID: 3BOM) and its ligand (PDB ID: HEM) have been elucidated, and the interacting residues have been clearly identified. It has been emphasized that the amino acid and water residues MET32, TYR42, PHE43, HIS45, TRP46, HIS59, THR62, ILE63, GLN66, ILE67, LEU84, LEU87, LEU92, VAL94, ASN98, PHE99, LEU102, LEU133, LEU137, HOH210, HOH225, HOH246, HOH251, HOH263, HOH288, HOH301, HOH318, HOH319, HOH321, HOH343, and HOH345 are significant in terms of interaction (https://www.ebi.ac.uk/pdbe/entry/pdb/3bom?activeTab=ligands, accessed on 10 May 2026). In this study, it was determined that our ligand EA interacts with these areas of Hb in a similar way. It has also been observed that it forms an H-bond in the same way as LEU102 and pi-pi interactions with PHE99.

A limitation of the present study is that neither the actual Pb concentrations in the exposure water nor Pb accumulation in the examined tissues were measured. Nevertheless, nominal Pb concentrations were maintained using a semi-static exposure protocol under identical experimental conditions for all groups. In addition, biomarkers were evaluated at the individual fish level, and potential tank-level variability was not included as a separate factor in the statistical model. Therefore, the present findings provide limited information regarding Pb concentration dynamics in the exposure water, tissue-level Pb accumulation, and possible tank-related variability. Despite these limitations, the results indicate that dietary EA may exert protective effects against Pb-induced hematological, immunological, and oxidative stress responses. However, within the scope of the present study, whether these protective effects are primarily associated with reduced Pb bioaccumulation or with the attenuation of Pb-induced oxidative stress and related physiological responses remains to be clarified.

## 5. Conclusions

This study demonstrated that Pb exposure in *C. carpio* induces pronounced toxicity by disrupting hematological (RBC, Hb, Ht), immunological (WBC, NBT, PA, LYZ, IgM, MPO), and oxidative stress parameters. Pb exposure increased MDA and GST levels while significantly decreasing SOD, CAT, GSH-Px, and GSH, indicating a central role for oxidative stress in Pb-induced toxicity. In contrast, EA supplementation reduced lipid peroxidation, lowered MDA and GST levels, preserved GSH content, and enhanced SOD, CAT, and GSH-Px activities. Concurrent improvements in RBC, Hb, Ht, as well as WBC, NBT, PA, LYZ, IgM, and MPO further demonstrate the antioxidant and immunomodulatory effects of EA. Molecular docking analysis indicated that EA exhibited micromolar binding affinities toward both Hb and IgM. Among the two targets, Hb showed a more favorable docking score, whereas IgM formed a greater number of hydrogen-bond interactions with EA. Docking analyses suggest that EA exhibits micromolar-level interactions within both molecules (Hb, IgM), and when comparing their affinities, IgM has a better binding score and formed more bonds in terms of hydrogen bond interactions. However, the significance of these interactions remains unclear, as the active site and important interactions for IgM have not been previously established with crystal ligands. In contrast, EA is located quite close to HEM on HB, showing similar and significant interactions. Overall, EA markedly alleviated Pb-induced oxidative stress and immunosuppression, suggesting its potential as a functional dietary additive to enhance fish resilience to heavy metal stress in aquaculture.

## Figures and Tables

**Table 1:** Molecular docking scores, interaction types, and estimated inhibition constants of EA on Hb and IgM.

Macromolecule /Pdb ID	Autodock Results			Vina Results
	Interacting Residues (Types)	Estimated Inhibition Constant, Ki	Best Docking Score	Best Docking Score
Hb /3BOM	PHE99 (Pi-Pi) LEU102 (H-Bond)	10.66 µM	−6.78	−8.8
IgM /8GHZ	A:ASN374 (H-Bond) B:ASP345 (H-Bond)	9.79 µM	−6.83	−9.1

µM: micromolar, Docking Score: kcal/mol.

## Data Availability

The data presented in this study are available from the corresponding author upon reasonable request.
